# Differential Effects of Polyethylene and Polystyrene Microplastics on the Composition and Functional Attributes of the Oat (*Avena sativa* L.) Rhizosphere Microbiome

**DOI:** 10.3390/plants15152403

**Published:** 2026-08-06

**Authors:** Lingping Zhao, Zhibo Yang, Wanqiao Zhang, Qunying Wang, Shitu Tan, Pei Mao, Wenfeng Ma

**Affiliations:** College of Animal Science and Technology, Henan University of Science and Technology, Luolong District, Kaiyuan Avenue, No. 263, Luoyang 471023, China; yzb20000102@163.com (Z.Y.); zwq1630720@163.com (W.Z.); 15081751970@163.com (Q.W.); t_s_t@sina.com (S.T.); maop17@163.com (P.M.); a113boy@163.com (W.M.)

**Keywords:** polyethylene, polystyrene, bacterial community, functional gene

## Abstract

Microplastics (MPs) are increasingly accumulating in agricultural soils, while their effects on crop-associated rhizosphere microbial communities and ecological functions remain insufficiently understood, particularly for different polymer types. In this study, polyethylene (PE) and polystyrene (PS) microplastics with the same particle size (2 μm) were applied at different concentrations (0, 0.1%, 0.5%, 1%, and 5%, *w*/*w*) to investigate their effects on oat rhizosphere bacterial communities and predicted functional potentials. The results showed that microplastic addition significantly altered bacterial community diversity, composition, and predicted functional profiles. Compared to the control (Ctrl), the 1% PE treatment significantly reduced bacterial richness-related indices (*p* < 0.05), whereas PS mainly affected bacterial diversity. Microplastic treatments also reshaped dominant bacterial taxa and altered the predicted functional potentials associated with carbon and nitrogen cycling. In particular, the 1% PE treatment significantly reduced the predicted relative abundance of the carbon fixation-related gene *cbbL* (*p* < 0.05). In addition, high-concentration (5%) PE enhanced several predicted functional potentials related to nitrogen cycling, including *nifH*, *ureA*, and *amoC*. Overall, PE and PS microplastics induced polymer- and concentration-dependent changes in oat rhizosphere bacterial communities. These findings suggest that microplastic accumulation in agricultural soils may influence ecosystem processes by modifying microbial diversity and potential biogeochemical functions. This study provides new insights into the ecological consequences of different microplastic polymers in crop rhizosphere ecosystems and highlights the importance of considering polymer-specific effects when evaluating soil microplastic pollution.

## 1. Introduction

The extensive production and widespread application of plastic products have resulted in the continuous accumulation of plastic residues in terrestrial and aquatic ecosystems [1]. Through weathering, ultraviolet radiation, mechanical abrasion, and biodegradation processes, large plastic debris can gradually fragment into microplastics (MPs), thereby altering their physicochemical properties and environmental behaviors. Microplastics are generally defined as plastic particles smaller than 5 mm in diameter [2,3]. Due to their small size, persistence, large specific surface area, and potential ecological risks, MPs have been recognized as emerging global pollutants of increasing environmental concern [4]. Among different environmental compartments, agricultural soils represent an important sink for MPs because of the long-term application of plastic mulching films, organic fertilizers, sewage sludge, irrigation practices, and atmospheric deposition [5,6,7]. Unlike aquatic environments, agricultural soils provide direct interfaces among MPs, plant roots, soil particles, and microorganisms [8], suggesting that microplastic contamination may have profound consequences for crop production and soil ecosystem functioning.

Soil microorganisms are essential regulators of terrestrial ecosystem processes, including organic matter decomposition, nutrient transformation, and biogeochemical cycling [9]. Increasing evidence indicates that MPs can modify soil microbial communities through multiple direct and indirect pathways. Physically, MPs can alter soil aggregation, porosity, bulk density, and water-holding capacity, thereby modifying microbial habitats and ecological niches [10]. The large surface area and hydrophobic characteristics of MPs also provide potential attachment sites for microbial colonization and biofilm formation [11], which may influence microbial interactions and community assembly. Chemically, MPs can adsorb nutrients, organic contaminants, and other environmental pollutants, affecting microbial exposure and metabolic processes [12]. Furthermore, additives released from plastic polymers during aging and degradation may induce physiological stress and alter microbial functions [13]. In plant–soil systems, MPs may indirectly regulate rhizosphere microbial communities by affecting plant growth, root morphology, and root exudate composition, which subsequently influence microbial recruitment and nutrient availability [14]. Previous studies have demonstrated that MPs can reduce microbial biomass, inhibit enzyme activities associated with carbon and nitrogen cycling, and reshape bacterial diversity and community composition [15,16,17]. However, other studies have reported that certain MPs, particularly biodegradable polymers, may provide additional carbon sources or microbial colonization surfaces and stimulate microbial activity under specific environmental conditions [18,19]. These inconsistent responses suggest that the ecological effects of MPs are highly dependent on polymer type, environmental conditions, and microbial functional characteristics.

Different types of plastic polymers may induce distinct ecological effects because of their contrasting physicochemical properties. Polyethylene (PE) and polystyrene (PS) are among the most frequently detected microplastics in terrestrial environments and represent two important polymer types associated with agricultural and domestic plastic waste [20,21]. PE is extensively used in agricultural plastic films and exhibits high chemical stability and environmental persistence, whereas PS contains aromatic structures and possesses different surface properties that may influence pollutant adsorption, microbial attachment, and ecological toxicity [22]. These polymer-specific characteristics may result in different interactions with soil microorganisms and subsequently alter microbial community composition, metabolic activities, and ecosystem functions [23]. Soil microorganisms play central roles in regulating carbon and nitrogen cycling through processes such as organic carbon transformation, carbon fixation, methane metabolism, nitrogen fixation, and nitrogen transformation. Previous studies have demonstrated that microplastics can affect microbial functional genes involved in carbon degradation, carbon fixation, methane metabolism, nitrification, denitrification, and nitrogen fixation by reshaping microbial communities and metabolic pathways [24,25,26]. However, how different microplastic polymers, particularly PE and PS, regulate rhizosphere microbial communities and their potential functional responses associated with carbon and nitrogen cycling remains insufficiently understood, especially in crop-associated soil ecosystems.

Oat is an important cereal and forage crop with broad environmental adaptability and ecological significance in agricultural systems [27]. Compared to major crops such as wheat and rice, the responses of oat rhizosphere microbial communities to microplastic contamination remain relatively unexplored. In addition, oats possess a well-developed root system that establishes complex interactions with rhizosphere microorganisms, making them a suitable model for investigating plant–microbe responses under environmental stress conditions. Understanding how MPs affect oat rhizosphere ecosystems is therefore important for evaluating the potential impacts of plastic contamination on agricultural sustainability.

Although previous studies have investigated the effects of MPs on soil microbial communities, several knowledge gaps remain. First, most existing studies have focused on bulk soil microbial communities, whereas the responses of rhizosphere bacterial communities under microplastic contamination remain insufficiently understood. Second, the comparative effects of different polymer types, particularly PE and PS, on rhizosphere bacterial community composition and potential functional profiles require further investigation. Third, the impacts of MPs on microbial functional potentials related to carbon and nitrogen cycling in crop rhizosphere soils remain insufficiently characterized. In this study, environmentally relevant concentrations of MPs (0.1%, 0.5%, and 1%, *w*/*w*) were selected based on previous studies, while a higher concentration (5%) was included as an extreme contamination scenario to evaluate potential microbial responses under severe microplastic accumulation conditions. Therefore, this study aimed to investigate the effects of PE and PS microplastics at different contamination levels on oat rhizosphere bacterial diversity, community composition, and predicted functional profiles associated with carbon and nitrogen cycling. We hypothesized that PE and PS microplastics would exert differential effects on oat rhizosphere bacterial communities due to their distinct physicochemical properties, and microplastic-induced shifts in bacterial communities would alter predicted microbial functions related to carbon and nitrogen cycling. This study provides new insights into the responses of rhizosphere bacterial communities to microplastic contamination and improves our understanding of microbial ecological consequences in agricultural soils.

## 2. Results

### 2.1. Effects of Microplastic Treatments on Alpha and Beta Diversity

**Figure 1:**
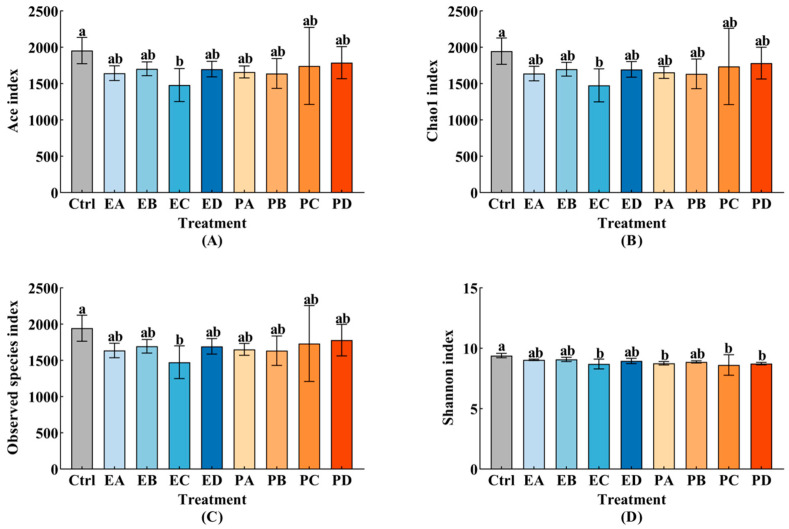
Effects of polyethylene (PE) and polystyrene (PS) microplastics on the alpha diversity of rhizosphere bacterial communities. (**A**) Ace index, (**B**) Chao1 index, (**C**) Observed species index, (**D**) Shannon index. Community richness was evaluated using the Ace, Chao1, and Observed species indices, while community diversity was assessed using the Shannon index. A one-way ANOVA was conducted, followed by a Duncan’s post hoc test. Lowercase letters above bars indicate significant differences (*p* < 0.05). Ctrl, control group; EA, EB, EC, and ED represent PE treatments at 0.1%, 0.5%, 1%, and 5%, respectively; PA, PB, PC, and PD represent PS treatments at 0.1%, 0.5%, 1%, and 5%.

For clarity, the PE treatments were designated as EA, EB, EC, and ED, corresponding to 0.1%, 0.5%, 1%, and 5% PE, respectively, whereas the PS treatments were designated as PA, PB, PC, and PD, corresponding to 0.1%, 0.5%, 1%, and 5% PS, respectively. Ctrl served as the control treatment. As shown in Figure 1, compared with the Ctrl group, the abundance-based coverage estimator (ACE), Chao1, and observed species indices of the bacterial community were significantly inhibited under the EC group (*p* < 0.05), whereas PS stress had no significant effects on these indices (*p* > 0.05). In terms of bacterial diversity, the Shannon index reached its lowest value under the PC group. The EC group significantly reduced the Shannon index (*p* < 0.05), while no significant differences were observed among the PE treatment groups (*p* > 0.05).

**Figure 2:**
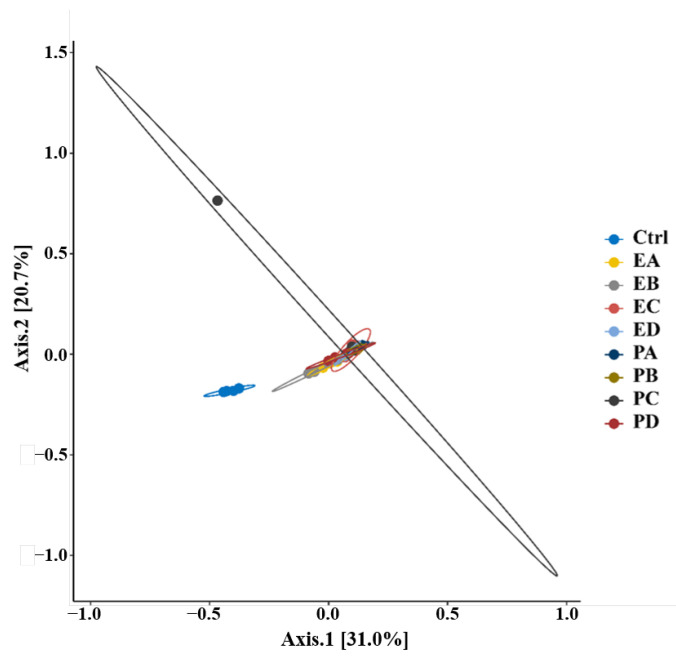
Principal coordinates analysis (PCoA) of the beta diversity of rhizosphere bacterial communities under polyethylene (PE) and polystyrene (PS) microplastic treatments. PCoA based on rarefied amplicon sequence variant (ASV) abundances was used to depict patterns of bacterial beta diversity. Ctrl, control group; EA, EB, EC, and ED represent PE treatments at 0.1%, 0.5%, 1%, and 5%, respectively; PA, PB, PC, and PD represent PS treatments at 0.1%, 0.5%, 1%, and 5%.

The first principal coordinate (PC1) and the second principal coordinate (PC2) explained 31.0% and 20.7% of the total variation, respectively (Figure 2). The beta diversity analysis revealed differences in bacterial community composition among treatments. Briefly, the bacterial community of the Ctrl was separated from those of the microplastic-treated groups, whereas substantial overlap was observed between the PE and PS treatment groups. Although most treatments clustered closely together, the PC group was relatively distinct from the other groups. In addition, analysis of similarities (ANOSIM) and permutational multivariate analysis of variance (PERMANOVA) were used to examine the significance of differences in the beta diversity of soil bacterial communities among different microplastic treatments (Table 1). The results showed that, with the exception of the EA and EB, EB and PD, EC and PC, EC and PD, PA and PC, and PC and PD groups, there were significant differences in soil bacterial community composition between all other pairs of treatment groups (*p* < 0.05).

### 2.2. Effects of Microplastic Treatments on the Composition of Rhizosphere Soil Bacterial Communities

**Figure 3:**
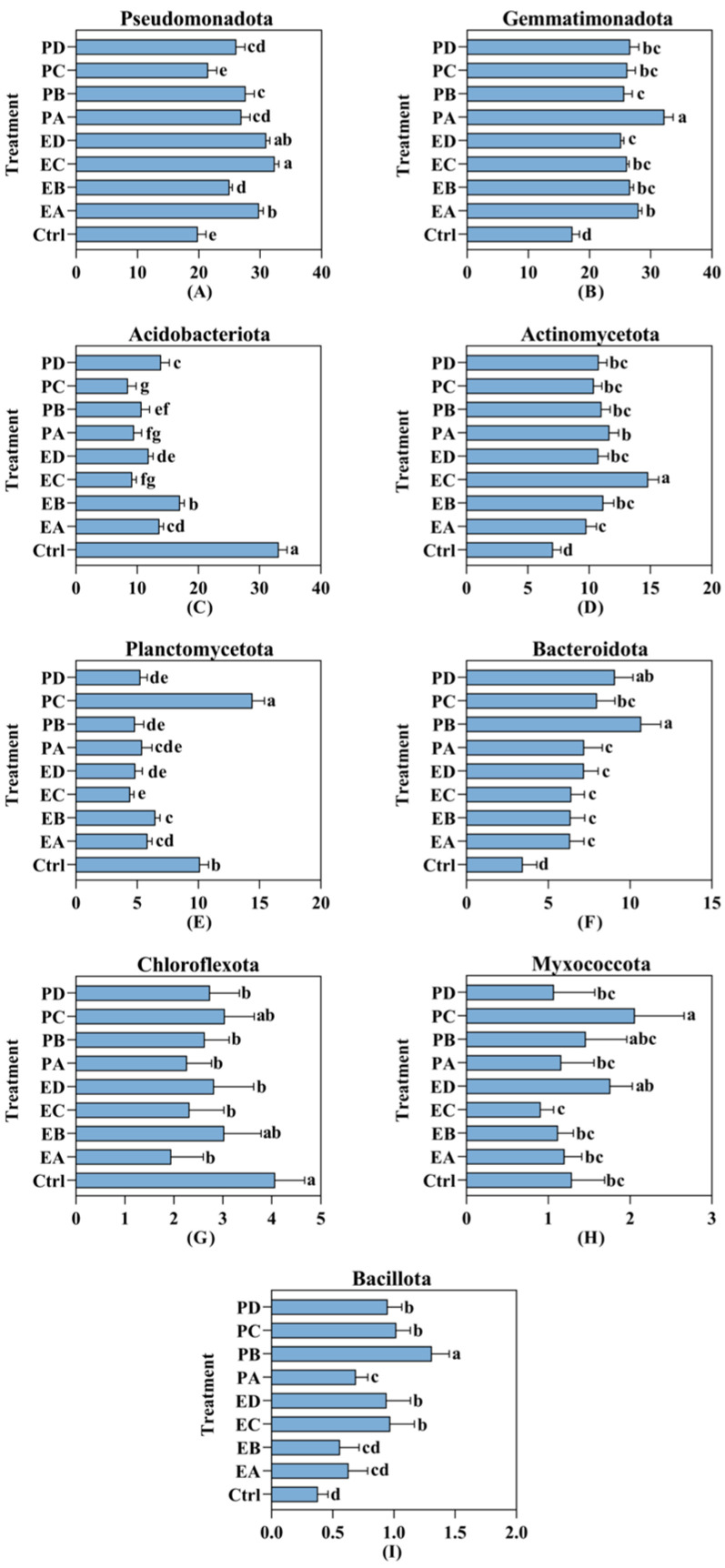
Relative abundances of the dominant bacterial phyla in oat rhizosphere soil under polyethylene (PE) and polystyrene (PS) microplastic treatments. (**A**) Pseudomonadota; (**B**) Gemmatimonadota; (**C**) Acidobacteriota; (**D**) Actinomycetota; (**E**) Planctomycetota; (**F**) Bacteroidota; (**G**) Chloroflexota; (**H**) Myxococcota; (**I**) Bacillota. A one-way ANOVA was conducted, followed by a Duncan’s post hoc test. Lowercase letters above bars indicate significant differences (*p* < 0.05). Ctrl, control group; EA, EB, EC, and ED represent PE treatments at 0.1%, 0.5%, 1%, and 5%, respectively; PA, PB, PC, and PD represent PS treatments at 0.1%, 0.5%, 1%, and 5%.

Compared to the Ctrl, the EC group significantly promoted the relative abundances of Pseudomonadota and Actinomycetota, both of which reached their highest values under this treatment (*p* < 0.05). The relative abundance of Acidobacteriota in the Ctrl was significantly higher than that in the PE and PS microplastic treatments (*p* < 0.05). The relative abundance of Bacteroidota significantly increased with increasing PE microplastic concentration (*p* < 0.05), although no significant differences were observed among the PE treatment groups (*p* > 0.05). Under the PA group, Gemmatimonadota showed the highest relative abundance, whereas the relative abundances of Bacteroidota and Bacillota reached their highest levels under the PB group. In addition, the PC group significantly promoted the relative abundances of Planctomycetota and Myxococcota (*p* < 0.05), both of which exhibited their highest relative abundances under this treatment (Figure 3).

**Figure 4:**
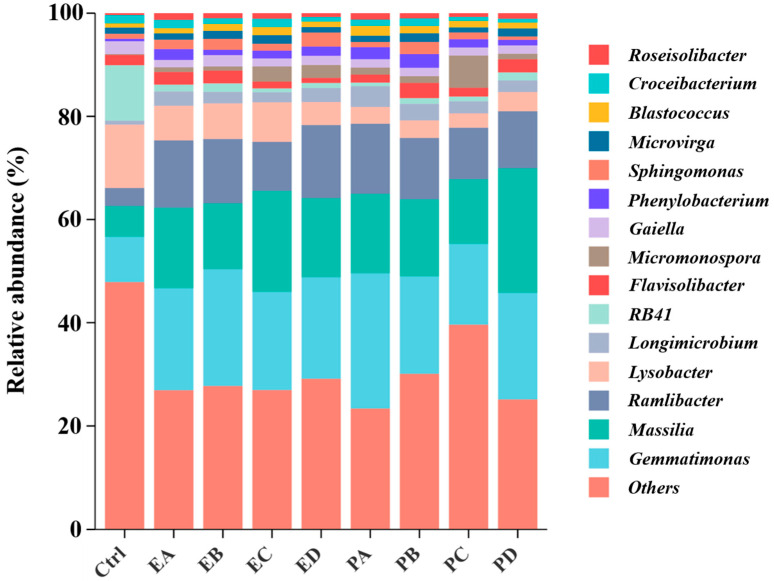
Relative abundances of the dominant bacterial genera in oat rhizosphere soil under polyethylene (PE) and polystyrene (PS) microplastic treatments. Ctrl, control group; EA, EB, EC, and ED represent PE treatments at 0.1%, 0.5%, 1%, and 5%, respectively; PA, PB, PC, and PD represent PS treatments at 0.1%, 0.5%, 1%, and 5%.

Compared to the Ctrl, the relative abundance of *Gemmatimonas* reached its highest level in the PA group, whereas *Massilia* exhibited the highest relative abundance in the PD group. The relative abundance of *Ramlibacter* reached its maximum under the 5% PE microplastic treatment, while the 1% PS microplastic treatment inhibited *Lysobacter* and reduced its relative abundance to the lowest level. In addition, *Longimicrobium* and *Flavisolibacter* showed their highest relative abundances in the PB group (Figure 4).

### 2.3. Linear Discriminant Analysis Effect Size (LEfSe) Analysis of Differential Bacterial Taxa in Oat Rhizosphere Soil Under Microplastic Treatments

**Figure 5:**
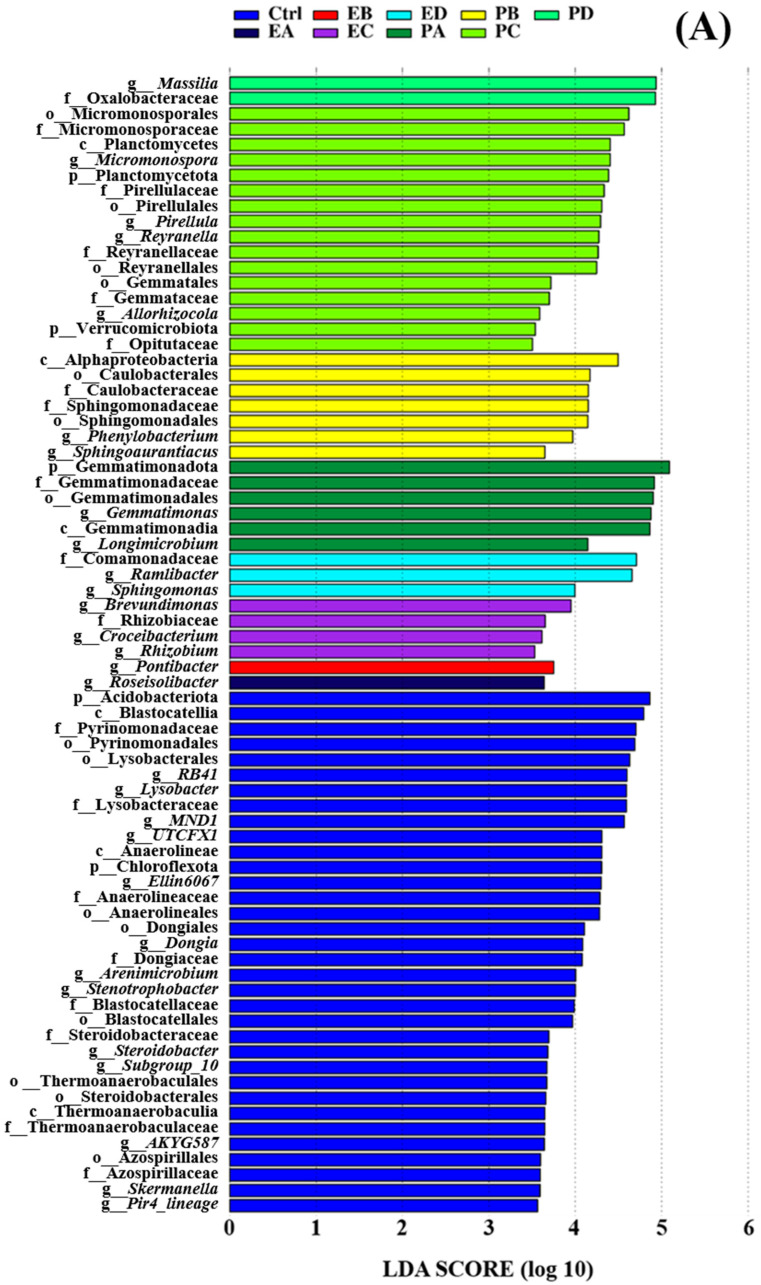

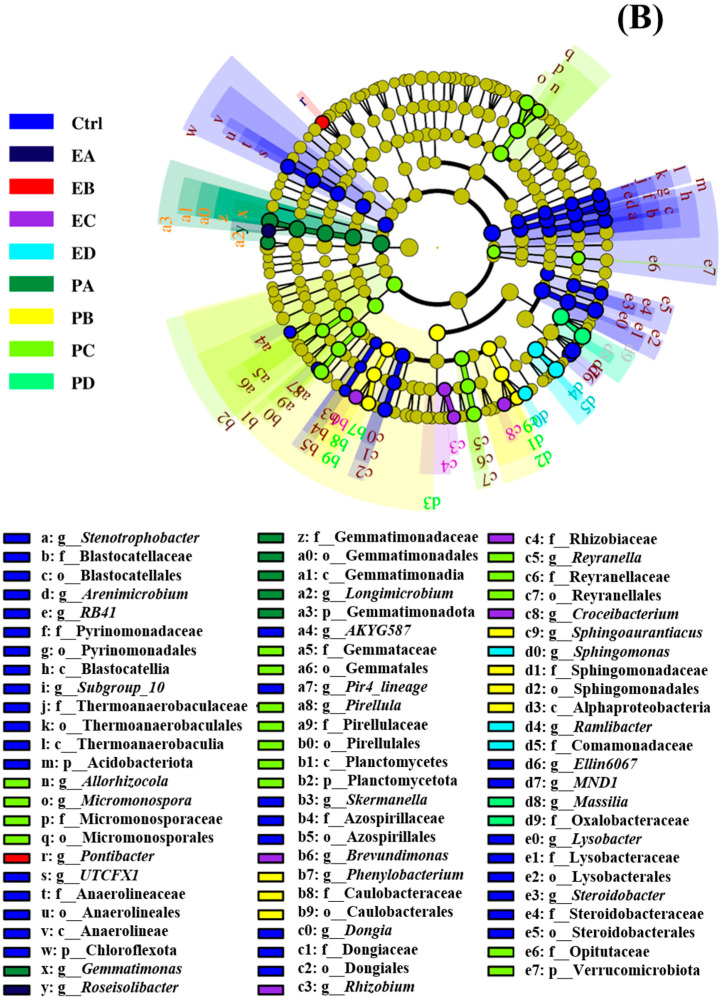
Linear discriminant analysis effect size (LEfSe) analysis of rhizosphere bacterial communities in oats under polyethylene (PE) and polystyrene (PS) microplastic treatments ((**A**) is the LDA value distribution map; (**B**) is the evolutionary branch diagram). Ctrl, control group; EA, EB, EC, and ED represent PE treatments at 0.1%, 0.5%, 1%, and 5%, respectively; PA, PB, PC, and PD represent PS treatments at 0.1%, 0.5%, 1%, and 5%.

The results of the linear discriminant analysis of bacterial community composition under different microplastic treatments are presented in Figure 5, with an LDA threshold value greater than 3.5. *Lysobacter* and *Dongia* were significantly enriched in the Ctrl (*p* < 0.05). In the EA group, *Roseisolibacter* was significantly enriched, whereas *Pontibacter* showed significant enrichment in the EB group (*p* < 0.05). Under the EC group, *Brevundimonas* and *Croceibacterium* were significantly enriched, while *Ramlibacter* and *Sphingomonas* exhibited significant enrichment under the ED group (*p* < 0.05). In the PA group, the abundances of *Gemmatimonas* and *Longimicrobium* significantly increased. In the PB group, *Phenylobacterium* was significantly enriched. In the PC group, the abundances of *Micromonospora* and *Pirellula* significantly increased, whereas *Massilia* showed significant enrichment in the PD group (*p* < 0.05).

### 2.4. PICRUSt2 Functional Prediction of Bacterial Communities in Oat Rhizosphere Soil Under Microplastic Treatments

**Figure 6:**
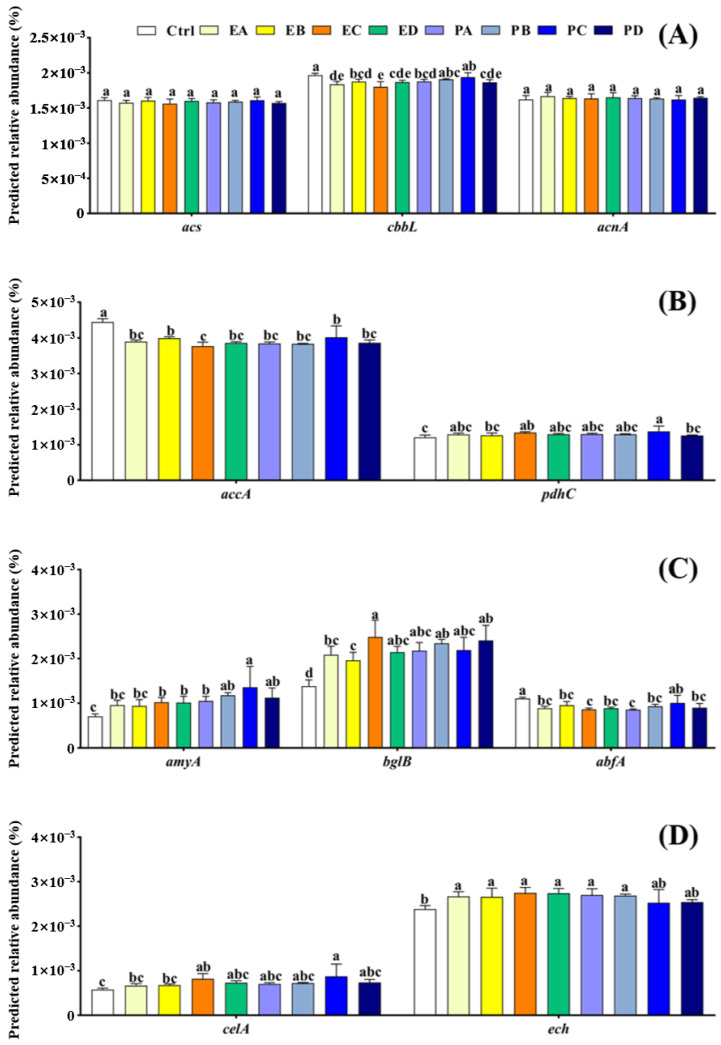
Effects of polyethylene (PE) and polystyrene (PS) microplastics on the predicted abundance of genes involved in carbon fixation (**A**,**B**) and carbon degradation (**C**,**D**). A one-way ANOVA was conducted, followed by a Duncan’s post hoc test. Lowercase letters above bars indicate significant differences (*p* < 0.05). Ctrl, control group; EA, EB, EC, and ED represent PE treatments at 0.1%, 0.5%, 1%, and 5%, respectively; PA, PB, PC, and PD represent PS treatments at 0.1%, 0.5%, 1%, and 5%.

**Figure 7:**
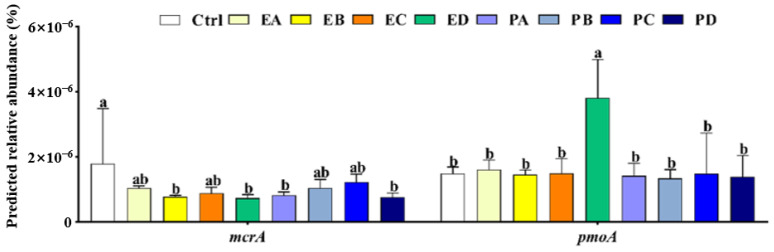
Effects of polyethylene (PE) and polystyrene (PS) microplastics on the predicted abundance of methane metabolism-related genes. A one-way ANOVA was conducted, followed by a Duncan’s post hoc test. Lowercase letters above bars indicate significant differences (*p* < 0.05). Ctrl, control group; EA, EB, EC, and ED represent PE treatments at 0.1%, 0.5%, 1%, and 5%, respectively; PA, PB, PC, and PD represent PS treatments at 0.1%, 0.5%, 1%, and 5%.

This study mainly focused on predicted functional genes with relatively high abundances. For carbon fixation-related genes (Figure 6), the predicted abundances of *acs* and *acnA* did not change significantly after PE and PS microplastic treatments (*p* > 0.05), whereas the predicted *accA* gene abundance significantly decreased (*p* < 0.05). Compared to the Ctrl group, the EA, EB, EC, and ED groups significantly inhibited the predicted *cbbL* gene abundance (*p* < 0.05), with reductions of 6.57%, 4.63%, 8.31%, and 4.97%, respectively, and the lowest value was observed under the EC group. For the *abfA* gene, its predicted abundance decreased to the lowest level under the PA group. During methane metabolism (Figure 7), compared to the Ctrl group, the EB, ED, PA, and PD groups significantly reduced the predicted *mcrA* gene abundance (*p* < 0.05). The ED group significantly increased the predicted *pmoA* gene abundance (*p* < 0.05), with an increase of 156.21% compared to the Ctrl group, whereas the remaining treatment groups did not exert significant effects on predicted *pmoA* gene abundance (*p* > 0.05).

**Figure 8:**
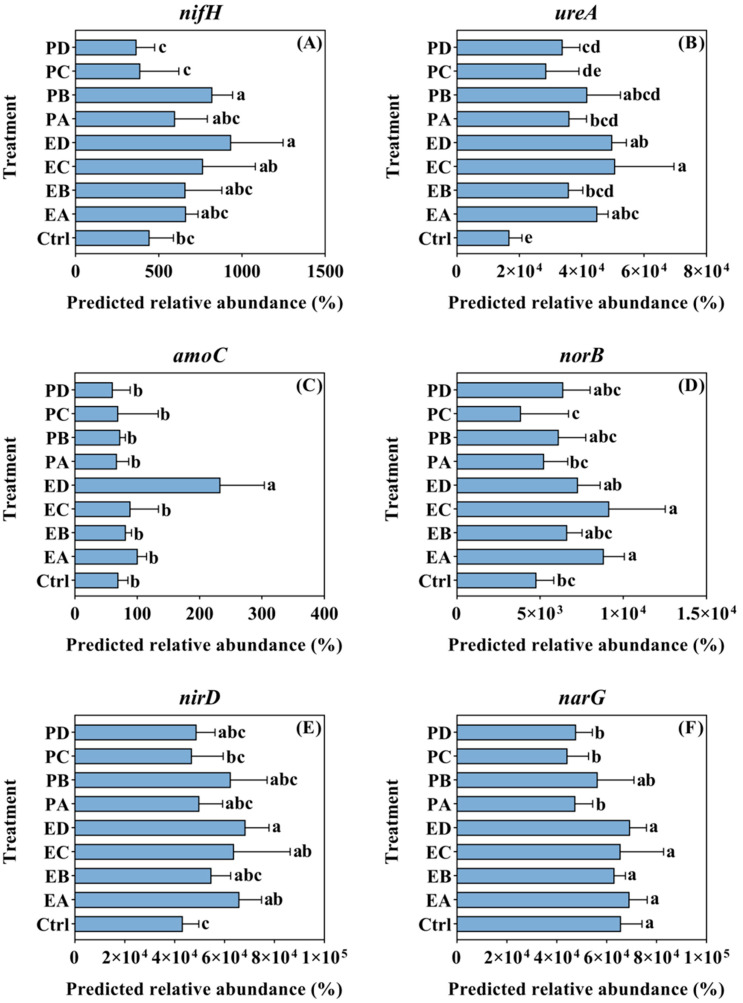
Effects of polyethylene (PE) and polystyrene (PS) microplastics on the predicted abundance of genes involved in the nitrogen cycle (**A**–**F**). A one-way ANOVA was conducted, followed by a Duncan’s post hoc test. Lowercase letters above bars indicate significant differences (*p* < 0.05). Ctrl, control group; EA, EB, EC, and ED represent PE treatments at 0.1%, 0.5%, 1%, and 5%, respectively; PA, PB, PC, and PD represent PS treatments at 0.1%, 0.5%, 1%, and 5%.

Among the genes related to the nitrogen fixation process (Figure 8), the ED group significantly increased the predicted *nifH* gene abundance associated with nitrogen fixation (*p* < 0.05), and its abundance increased by 109.93% compared to that of the Ctrl group. For genes associated with the ammonia oxidation process, all microplastic treatments except the PC group significantly affected the predicted *ureA* gene abundance (*p* < 0.05). Moreover, the predicted *ureA* gene abundances in the high-concentration PE microplastic treatment groups (EC and ED) were higher than those in the PS microplastic treatment groups (PC and PD). With respect to the nitrification process, the variation trend of the predicted *amoC* gene abundance was consistent with that of the predicted *nifH* gene abundance during nitrogen fixation, and both genes significantly increased under the ED group (*p* < 0.05). Among the functional genes involved in the denitrification process, the EA and EC groups significantly enhanced the predicted *norB* gene abundance (*p* < 0.05), with increases of 84.53% and 91.47%, respectively, compared with the Ctrl group, whereas the PS microplastic treatment groups did not significantly affect predicted *norB* gene abundance (*p* > 0.05). During assimilatory nitrate reduction and dissimilatory nitrate reduction, the PS microplastic treatments did not significantly affect the predicted *nirD* gene abundance, and the PE microplastic treatments did not significantly influence the predicted *narG* gene abundance (*p* > 0.05). However, the EA, EC, and ED groups significantly increased the predicted *nirD* gene abundance, whereas the PA, PC, and PD groups significantly inhibited the predicted *narG* gene abundance (*p* < 0.05).

## 3. Discussion

The Shannon index reflects community diversity, and the ACE, Chao1, and observed species indices reflect species richness [28]. In this study, 0.1% and 0.5% PE treatments did not significantly affect these indices, suggesting that bacterial communities may have maintained relative stability under relatively low PE exposure levels. This result may indicate that soil microorganisms possess a certain degree of resistance or adaptability to mild microplastic stress, allowing community diversity and richness to remain relatively unchanged within a limited exposure range [29]. Therefore, at relatively low addition levels (0.1% and 0.5%), PE appeared to have limited effects on bacterial α-diversity in the oat rhizosphere and did not induce obvious changes in bacterial richness and diversity [30]. In contrast, 1% PE significantly reduced species richness and diversity, indicating that higher PE concentrations were associated with stronger disturbances to bacterial community structure [31]. PE microplastics may influence soil physicochemical properties, including soil structure and porosity, which could subsequently affect water and nutrient availability and microbial habitats [32]. They may also interact with surrounding organic compounds and modify the soil microenvironment [33,34]. In addition, potential transformation products or surface-associated substances of microplastics have been suggested to influence microbial communities [35]. These possible mechanisms may contribute to shifts in environmentally sensitive bacterial taxa and consequently affect community richness and diversity [36,37]. Previous studies have demonstrated that PS microplastics reduce the Shannon index [38]. Similarly, 0.1%, 1%, and 5% PS treatments significantly reduced the Shannon index, suggesting that PS exposure was associated with decreased bacterial diversity in the present study. However, PS treatments did not significantly affect the ACE, Chao1, or observed species indices, whereas the 1% PE treatment significantly reduced species richness indices. This difference suggests that bacterial responses may depend on microplastic characteristics and environmental conditions [23,39]. Previous reports indicated that polylactic acid (PLA) treatment did not significantly alter bacterial richness or diversity [30], and soil bacterial α-diversity did not change significantly after 90 days of contamination with microplastics of different types and concentrations [40], suggesting that variations among studies may be related to differences in soil properties, microplastic types, particle characteristics, addition concentrations, cultivation conditions, and exposure durations. Soil texture and physicochemical characteristics can influence the adsorption, mobility, and ecological effects of microplastics by regulating their interactions with soil particles and microorganisms. In the present study, the same soil type and texture were used across all treatments, which minimized the influence of soil heterogeneity on treatment comparisons. Therefore, the observed differences among treatments were mainly associated with microplastic addition rather than variations in soil properties.

Microplastic addition was associated with changes in microbial community structure, with the specific effects depending on microplastic type, addition concentration, and cultivation duration. Previous studies demonstrated that non-biodegradable microplastics, including PE and PS, could influence soil bacterial community structure [41], consistent with the present study. The results showed that at 5% addition, both PE and PS markedly changed the composition and relative abundances of major bacterial phyla compared with the control. Pseudomonadota accounted for a high proportion in all treated soils, and all PE treatments significantly increased its relative abundance, possibly because Pseudomonadota members were Gram-negative bacteria that were more likely to accumulate in the rhizosphere and played important roles in organic matter degradation [42,43]. In addition, Pseudomonadota generally preferred relatively loose soils, and PE microplastic may influence soil bulk density [19]. Furthermore, because PE microplastics mainly consisted of carbon, they might change soil carbon source structure and forms, providing new carbon sources for certain bacterial taxa and increasing the relative abundance of Pseudomonadota [39,44]. At the 5% addition level, the relative abundance of this phylum was significantly higher in PE- than PS-treated soil, indicating greater bacterial community sensitivity to PE microplastics, possibly due to PE’s functional groups facilitating aging in soil with stronger effects at high concentrations [45]. Although PS microplastics exhibited relatively low degradability, they may provide habitats for soil microorganisms, increasing the abundance of bacterial taxa involved in their degradation and ultimately altering microbial community structure [46,47,48], which is partially supported by our finding that all PS treatments significantly reduced Acidobacteriota relative abundance compared with the control. At the genus level, *Gemmatimonas* was the dominant genus across all treatments, and both PE and PS treatments significantly increased its abundance, with similar trends for other dominant genera. *Gemmatimonas* has been reported to be involved in soil phosphorus transformation processes. During growth and reproduction, this genus may produce metabolites that promote the dissolution and transformation of insoluble phosphates, potentially increasing soil available phosphorus and providing accessible phosphorus for plant growth [49].

Microbial carbon fixation in soil plays an important role in maintaining soil carbon sink functions and regulating the global carbon cycle, with the predicted abundance of carbon fixation-related functional genes commonly used as an indicator of potential carbon sequestration capacity. The study investigated the effects of different microplastic types and concentrations on soil carbon fixation by analyzing the predicted abundances of key carbon fixation-related genes, including *cbbL* and *accA*, and found that microplastic addition exerted varying degrees of interference depending on type and concentration. The *cbbL* gene, a key marker gene involved in the Calvin cycle and encoding the large subunit of ribulose-1,5-bisphosphate carboxylase/oxygenase, is widely used to indicate the potential capacity of microbial autotrophic carbon fixation. Previous studies have demonstrated that pollutants alter the abundance of *cbbL*-associated genes by affecting microbial metabolism or community structure. For example, Qian et al. [50] reported that agricultural plastic mulch decreased the abundance of carbon fixation-related genes, indicating a potential reduction in microbial carbon fixation capacity. In this study, PE treatment generally reduced the predicted *cbbL* gene abundance, consistent with previous findings. Related studies suggested that PE microplastic addition altered specific soil microbial communities and promoted organic carbon decomposition, reducing microbial demand for inorganic carbon fixation and the predicted relative abundance of carbon fixation-related genes [51]. Zhang et al. [52] found that microplastics weakened soil carbon sequestration potential by altering soil microbial communities, and the present results supported this view to a certain extent. In contrast, different PS concentrations showed different effects on the predicted *cbbL* gene abundance, with relatively small changes and even slight increases at 1% concentration, possibly due to adaptive microbial responses to environmental stress. Previous studies suggested that under low or moderate pollution, microorganisms upregulated certain metabolic pathways to maintain energy supply, increasing the predicted relative abundance of related functional genes [53]. However, when pollutant concentrations further increased, this adaptive mechanism might be disrupted, ultimately leading to the inhibition of metabolic functions. In addition to the Calvin cycle, other carbon fixation pathways were also influenced by environmental factors. The *accA* gene participated in fatty acid synthesis and certain autotrophic metabolic pathways, and its predicted abundance partly reflected microbial carbon assimilation potential. In this study, microplastic treatments generally reduced the predicted *accA* gene abundance, suggesting that microplastics might inhibit related carbon fixation pathways. Microplastics altered soil pore structure, water distribution, and nutrient availability, thereby affecting microbial growth and metabolism [54]. Furthermore, microplastic particles adsorbed organic matter and other pollutants, altering the microbial microenvironment and potentially reducing the predicted relative abundances of carbon fixation-related genes [55].

Soil organic carbon (SOC) degradation mainly depends on microbial-mediated organic matter decomposition, in which carbon degradation-related functional genes play important roles. The *abfA* gene is generally associated with hemicellulose degradation, and its predicted abundance partly reflected the potential for decomposition of unstable soil carbon components. Microplastics might thus alter soil carbon degradation by affecting the predicted functional potentials of microbial communities and metabolic activities. Studies indicated that microplastics could form new soil microenvironments and influence microbial growth and functional characteristics [56]. Under such conditions, the predicted abundances of carbohydrate degradation-related functional genes changed, affecting the decomposition of unstable organic carbon components such as hemicellulose. In addition, changes in soil enzyme activities may also serve as a mechanism by which microplastics indirectly influenced carbon degradation. For example, microplastics have been shown to increase phenol oxidase activity, which plays an important role in decomposing recalcitrant macromolecular organic compounds such as lignin, leading to the release of more dissolved organic carbon (DOC) into the soil solution [57]. DOC is readily utilized by soil microorganisms, promoting microbial respiration and CO_2_ production, which may accelerate SOC mineralization. Huang et al. [58] found that LDPE microplastics enhanced microbial carbohydrate degradation metabolism, further supporting that microplastics influence soil carbon cycling by regulating microbial metabolism. Previous studies have indicated that predicted *abfA* abundance may be associated with Actinomycetota, important soil hydrocarbon degraders involved in organic matter cycling through the degradation of aliphatic and aromatic hydrocarbons [59,60]. However, although Actinomycetota relative abundance increased under PE treatment, the predicted *abfA* gene abundance showed a decreasing trend, indicating that this gene was not entirely controlled by actinomycete abundance. A possible explanation is that microplastics may reduce the predicted functional potential or predicted abundance of *abfA*-carrying microorganisms by altering the soil microenvironment or affecting specific functional taxa.

Methane emissions from agricultural soils depend on the balance between methane production and consumption, jointly regulated by methanogens and methane-oxidizing bacteria [61,62,63]. When the functional potential associated with the methanogenic gene *mcrA* is high and that associated with the methane-oxidizing gene *pmoA* is low, methane production may increase while methane oxidation may be inhibited, potentially leading to elevated methane emissions [64]. However, inconsistent conclusions regarding microplastic effects on methane emissions have been reported, possibly reflecting different responses of methanogens and methane-oxidizing bacteria to different microplastic types. Han et al. [65] found that PET microplastics reduced methane emissions by increasing the predicted *pmoA* gene abundance, whereas PP microplastics did not significantly affect the predicted *mcrA* or *pmoA* gene abundances and consequently had no significant effect on methane emissions [64]. In this study, the 5% PE treatment significantly reduced the predicted *mcrA* gene abundance and significantly increased the predicted *pmoA* gene abundance, suggesting a reduced methane emission potential in soil. In contrast, PS microplastics did not significantly affect predicted methane oxidation-related functional genes and therefore might not have markedly altered soil methane emission potential. Furthermore, soil type also affected methane emissions. Microplastics significantly affected the predicted *mcrA* and *pmoA* gene abundances in soils from Jiangxi and Jiangsu Provinces but had no significant effects on predicted methane production- or oxidation-related functional genes in soils from Hunan and Zhejiang Provinces [52]. Collectively, these findings indicate that microplastic type and soil properties play critical roles in regulating agricultural soil methane production.

Microplastic pollution affects soil nitrogen cycling through multiple pathways [66]. Microplastics may inhibit nitrogen fixation by altering the soil environment and bacterial functions, potentially through reduced soil substrate respiration and altered soil physicochemical properties, thereby affecting the survival and activity of nitrogen-fixing microorganisms [67]. However, microplastics can also act as organic carbon sources, stimulating microbial activity and nitrogen mineralization, thereby increasing ammonium availability. They might also promote urea decomposition by stimulating urease activity or altering bacterial community structures favoring this pathway [68,69]. The present study showed that the 5% PE treatment significantly increased the predicted *nifH* and *ureA* gene abundances, suggesting enhanced nitrogen fixation and urea decomposition potentials. Several mechanisms may explain these effects. First, microplastics may release small carbon compounds during soil aging, providing additional energy and nutrients for ammonia-assimilating microorganisms and stimulating their growth and activity [70,71]. Sufficient carbon and nitrogen could promote bacterial growth, increasing bacterial abundance, as reflected by the significant increase in predicted *nifH* gene abundance [72,73,74]. Second, microplastics may improve soil environment by regulating aeration, moisture, and pH, creating more suitable habitats for nitrogen-fixing microorganisms. Particles of specific sizes may serve as microbial attachment sites, providing protection and promoting bacterial aggregation and proliferation [75,76,77]. Microplastics may also influence soil organic matter decomposition and nutrient fluxes, indirectly enriching nutrient resources for nitrogen-fixing microorganisms and enhancing nitrogen fixation [78,79]. Third, microplastics could interact with urea-degrading enzymes, altering their structure and enhancing their activity, thereby increasing the predicted *ureA* gene abundance [80], which accelerates bacterial urea decomposition and generates more carbon dioxide and ammonia while affecting soil nitrogen cycling. Furthermore, microplastics could alter bacterial community structure, increasing populations capable of efficient urea degradation and thereby enhancing predicted *ureA* abundance and urea decomposition rates [68,81]. Microplastics might also affect microbial metabolic pathways associated with *ureA*-related functional potential and urea decomposition. The 5% PS treatment significantly inhibited the predicted *narG* gene abundance and the predicted nitrate reduction potential. This inhibition mainly resulted from the interference of microplastics with bacterial physiological functions and environmental conditions through multiple mechanisms. Specifically, microplastics affected the predicted *narG* gene abundance, nitrate reductase activity, and bacterial growth through chemical interference and biological toxicity, thereby suppressing the normal physiological functions, gene expression, and activity of microorganisms expressing *narG*-associated functions [82,83]. In contrast, the 5% PS treatment did not significantly affect other nitrogen cycling-related predicted functional genes, including *amoC*, *norB*, and *nirD*. This may be due to the strong regulatory and compensatory capacities of complex genetic networks under environmental stress. By upregulating other key process-related genes and employing compensatory mechanisms, microorganisms might offset microplastic-induced inhibition, preventing significant impacts on overall nitrogen cycling [84].

Although this study demonstrated that microplastic addition altered rhizosphere microbial communities, the current experimental design cannot completely distinguish the direct effects of microplastics on soil microorganisms from indirect effects mediated by changes in plant growth and root-derived carbon inputs. Future investigations integrating plant-free soil incubation systems, root exclusion approaches, and root exudate measurements are needed to further clarify the relative contributions of direct microplastic–microbe interactions and plant-mediated rhizosphere responses under microplastic stress.

Furthermore, the limitations of PICRUSt2-based functional prediction should be considered. PICRUSt2 infers microbial functional potentials based on 16S rRNA gene sequencing data and reference genome databases; therefore, the predicted functional profiles may be affected by the completeness of reference genomes and do not necessarily represent actual gene abundance, transcriptional activity, or metabolic functions. Moreover, PICRUSt2 cannot capture post-transcriptional regulation and enzyme-level processes. Therefore, the predicted changes in carbon and nitrogen cycling-related functions in this study are regarded as potential functional responses rather than direct evidence of altered microbial processes and require further validation through molecular approaches such as quantitative PCR, metagenomics, or metatranscriptomics.

## 4. Materials and Methods

### 4.1. Experimental Materials

Oat was selected as the target crop because it is an important cereal crop with strong environmental adaptability and is widely cultivated in agricultural systems, making it a suitable model for evaluating plant responses to soil contaminants. Among the available oat cultivars, “Sweet Oat 60” was selected for the pot experiment and was provided by Luoyang Academy of Agriculture and Forestry Sciences. This cultivar was selected because of its high digestible fiber content and good adaptability. The yellow fluvo-aquic soil used in the pot experiments was collected from the experimental farm of Henan University of Science and Technology. The soil was classified as a silt loam soil. The basic physicochemical properties of the soil were as follows: pH 7.96, organic carbon content 10.91 g kg^−1^, alkali-hydrolyzable nitrogen content 79.03 mg kg^−1^, available phosphorus content 11.37 mg kg^−1^, readily available potassium content 226.8 mg kg^−1^, and available iron content 6.18 mg kg^−1^. In addition, no microplastic residues were detected in the collected soil samples before microplastic addition. After collection, the soil was naturally air-dried outdoors and passed through a 2 mm sieve before use.

**Figure 9:**
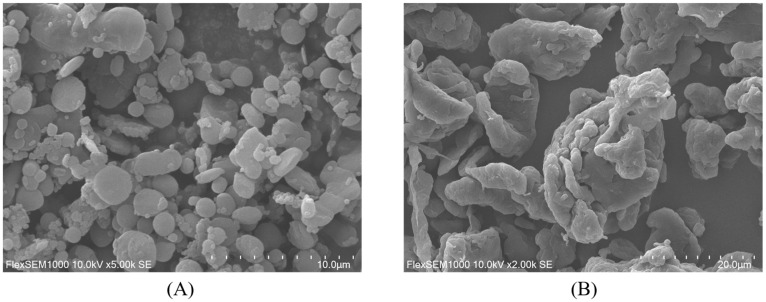
Characterization of microplastics. SEM images showing the morphology of PS (**A**) and PE (**B**).

Previous investigations have indicated that PE and PS microplastics were among the predominant types of microplastics detected in agricultural and grassland soils in China, with the majority of particles having diameters below 10 μm [85]. Based on these findings, PE and PS microplastics with an average particle size of 2 μm were chosen as representative materials to simulate microplastic contamination in agricultural soils, with the aim of evaluating their effects on bacterial community composition in the oat rhizosphere soil. The microplastics used in this experiment were primary microplastics, obtained directly from commercial 3D printing powders without additional additives (Dongguan Zhangmutou Suzhan Plastic Products Trading Department). The morphological characteristics of the PE and PS particles were examined by scanning electron microscopy (SEM). As shown in Figure 9, PS particles exhibited a relatively spherical shape, whereas PE particles showed irregular morphologies with sharp edges on their surfaces. The concentrations used in this study were consistent with those applied in our previous research [85] and were set at 0%, 0.1%, 0.5%, 1%, and 5% of soil weight (*w*/*w*), with the treatment without microplastic addition serving as the control (Ctrl). The concentrations of 0.1%, 0.5%, and 1% represented environmentally relevant microplastic contamination levels in agricultural soils. In addition, the higher concentration (5%) was included to evaluate microbial responses under potential severe microplastic contamination scenarios and to characterize concentration-dependent effects, as extreme contamination levels may occur locally due to long-term plastic accumulation. The required amounts of PE and PS microplastics were accurately weighed using a precision electronic balance (Sartorius, Göttingen, Germany) according to the designed concentrations and first mixed with a small amount of air-dried soil to obtain a homogeneous mixture. The premixed soil was then thoroughly blended with the remaining soil. Subsequently, the treated soils were allowed to equilibrate for 10 days prior to sowing, facilitating sufficient contact between microplastics and soil particles and improving the uniformity of microplastic distribution in the soil. Oat seeds with uniform grain size were selected and sterilized with 2% H_2_O_2_ solution for 30 min, after which they were rinsed several times with ultrapure water to remove residual disinfectant. Twenty seeds were sown in each pot, and each treatment included four replicates. A total of nine treatment groups were established in the experiment. The PE treatments were designated as EA (0.1% PE), EB (0.5% PE), EC (1% PE), and ED (5% PE) according to the concentration gradient, whereas the PS treatments were designated as PA (0.1% PS), PB (0.5% PS), PC (1% PS), and PD (5% PS). In addition, the Ctrl group was included. The experiment was arranged in a completely randomized block design and consisted of 36 pots in total. Ceramic pots were used in the pot experiment, with a top diameter of 20.5 cm, a height of 18.5 cm, and a bottom diameter of 14.5 cm, and each pot was filled with 4 kg of soil. The pot experiments were conducted under outdoor conditions in Luoyang City, Henan Province, China, from 30 September 2024 to 30 November 2024, with a duration of 60 days. During the experimental period, the average temperature was 18 °C and the average rainfall was 152.3 mm. Throughout the cultivation period, soil moisture was maintained by regularly monitoring soil conditions through visual inspection and manual checking. During the inspection, a small amount of surface soil was collected and gently touched or lightly squeezed by hand to assess its moisture status. When the soil appeared dry, loose, or lacked sufficient moisture based on these observations, water was manually added to maintain suitable moisture conditions and prevent drought stress. Weeds in the pots were regularly removed throughout the experimental period. At the end of the cultivation period, the oat plants were harvested. The shoots and roots were carefully separated, and the plant samples were collected for subsequent determination of growth and physiological parameters. The rhizosphere soil samples were collected by gently shaking the roots to remove loosely attached bulk soil, and the soil remaining on the root surfaces was considered as rhizosphere soil. The collected rhizosphere soil samples were immediately frozen in liquid nitrogen and then stored in a −80 °C freezer until bacterial DNA extraction and subsequent sequencing analysis.

### 4.2. Experimental Methods

#### 4.2.1. DNA Extraction and PCR Amplification

Genomic DNA was extracted from the rhizosphere soil samples using the CTAB method [86]. Subsequently, the purity and concentration of the extracted DNA were determined by 1% agarose gel electrophoresis. A 10 µL aliquot of the extracted DNA was transferred into centrifuge tubes, and the DNA concentration was adjusted to 1 ng/µL by dilution with sterile water to obtain the required genomic DNA template.

16S rRNA genes of distinct regions (16S V3-V4) were amplified used specific primer 341F (5′-CCTAYGGGRBGCASCAG-3′) and 806R (5′-GGACTACNNGGGTATCTAAT-3′) with the barcode [87]. All PCR reactions were carried out with 15 µL of Phusion^®^ High-Fidelity PCR Master Mix (New England Biolabs, Ipswich, MA, USA); 2 µM of forward and reverse primers, and about 10 ng template DNA. Thermal cycling consisted of initial denaturation at 98 °C for 1 min, followed by 30 cycles of denaturation at 98 °C for 10 s, annealing at 50 °C for 30 s, and elongation at 72 °C for 30 s. Finally, 72 °C for 5 min.

Mix same volume of 1XTAE buffer with PCR products and operate electrophoresis on 2% agarose gel for detection. PCR products were mixed in equidensity ratios. Then, mixture PCR products were purified with Universal DNA (TianGen, Beijing, China).

#### 4.2.2. Libraries Generated and Illumina Sequencing

Sequencing libraries were generated using NEB Next^®^ Ultra DNA Library Prep Kit (Illumina, San Diego, CA, USA) following manufacturer’s recommendations and index codes were added. The library quality was assessed on the Agilent 5400 (Agilent Technologies Co., Ltd., Santa Clara, CA, USA). At last, the library was sequenced on an Illumina platform and 250 bp paired-end reads were generated.

#### 4.2.3. Bioinformatics Analysis

The analysis was conducted by following the “Atacama soil microbiome tutorial” of Qiime2docs along with customized program scripts (https://docs.qiime2.org/2019.1/, 21 October 2025). Briefly, raw data FASTQ files were imported into the format which could be operated by QIIME2 system using qiime tools import program. Demultiplexed sequences from each sample were quality filtered and trimmed, de-noised, merged, and then the chimeric sequences were identified and removed using the QIIME2 dada2 plugin to obtain the feature table of amplicon sequence variant (ASV) [88]. The QIIME2 feature-classifier plugin was then used to align ASV sequences to a pre-trained GREENGENES 13_8 99% database to generate the taxonomy table [89]. Any contaminating mitochondrial and chloroplast sequences were filtered using the QIIME2 feature-table plugin. Based on the taxonomic classification information, the compositions of bacterial communities in oat rhizosphere soil under different treatments were analyzed [90]. At the feature sequence level, alpha diversity indices, including Observed OTUs, Chao1, ACE, and the Shannon index, were used to evaluate within-sample diversity [91]. Beta diversity indices based on Bray–Curtis distance were employed to assess differences in microbial community structure among samples, and the results were visualized through principal coordinates analysis (PCoA) [92]. In addition, the linear discriminant analysis effect size (LEfSe) method was applied to identify bacterial taxa with differential abundances among different groups and samples [93]. Finally, PICRUSt2 (version 2.3.0) was used to predict the potential functional profiles of the microbial communities based on KEGG Ortholog (KO) functional profiles [94]. The reference genome information used for functional prediction was obtained from the NCBI database. An NSTI threshold of 2 was applied during the prediction process, and sequences with NSTI values higher than this threshold were excluded to improve the reliability of functional predictions.

### 4.3. Data Analysis

The ACE, Chao1, Shannon, and observed species indices, dominant bacterial phyla relative abundances, as well as PICRUSt2-predicted functional gene abundance data, were subjected to statistical analysis. Before performing one-way analysis of variance (ANOVA), the normality of data distribution was evaluated using the Shapiro–Wilk test, and the homogeneity of variances was assessed using Levene’s test. All datasets met the assumptions of normality and homogeneity of variance. No data transformation was required before statistical analysis because all datasets satisfied the assumptions of ANOVA. Therefore, ANOVA followed by Duncan’s multiple range test was conducted using SPSS 23.0 software. Differences were considered statistically significant at *p* < 0.05. GraphPad Prism 9.0 software was used for graphical presentation of the data. PCoA was conducted to assess variations in microbial community structure among samples. Furthermore, LEfSe was employed to identify potential biomarkers across different taxonomic levels, and taxa with logarithmic LDA scores greater than 3.5 were regarded as significantly enriched.

## 5. Conclusions

This study provides new insights into the polymer-specific responses of oat rhizosphere bacterial communities to microplastic contamination by demonstrating that PE and PS microplastics induce distinct microbial responses across different contamination levels. Microplastic type and concentration altered bacterial community diversity and composition, with the 1% PE treatment significantly reducing bacterial richness indices compared with the control, indicating that different polymer types and exposure levels may differently influence rhizosphere microbial communities. Furthermore, PICRUSt2-based functional prediction suggested that microplastic exposure may modify microbial functional potentials associated with carbon and nitrogen cycling. Specifically, the 1% PE treatment significantly reduced the predicted relative abundance of the carbon fixation-related gene *cbbL*, whereas high-concentration (5%) PE enhanced several nitrogen cycling-related predicted functional potentials. However, these findings represent potential functional responses inferred from microbial community composition rather than directly measured microbial activities or actual gene abundances. Overall, this study advances the understanding of microplastic impacts on crop-associated rhizosphere ecosystems by highlighting the importance of polymer-specific effects in shaping microbial communities and their potential ecological functions. These findings contribute to the ecological risk assessment of microplastic contamination in agricultural soils by providing evidence that different plastic polymers may generate distinct microbial responses and potentially influence soil biogeochemical processes. Future studies integrating metagenomics, metatranscriptomics, quantitative PCR, and microbial enzyme activity measurements are needed to validate these predicted functional changes and further elucidate the mechanisms underlying microplastic–microbe interactions in soil ecosystems.

## Figures and Tables

**Table 1:** Non-parametric multivariate statistical analysis of the effects of polyethylene (PE) and polystyrene (PS) microplastic treatments on rhizosphere bacterial community composition.

Group	PERMANOVA		ANOSIM	
	*F*	*p*	*R*	*p*
Ctrl vs. EA	11.91	0.02	1.00	0.03
Ctrl vs. EB	8.82	0.03	1.00	0.03
Ctrl vs. EC	12.91	0.03	1.00	0.03
Ctrl vs. ED	15.84	0.02	1.00	0.04
Ctrl vs. PA	17.42	0.04	1.00	0.02
Ctrl vs. PB	18.42	0.03	1.00	0.04
Ctrl vs. PC	3.96	0.03	0.63	0.03
Ctrl vs. PD	10.88	0.03	1.00	0.02
EA vs. EB	1.62	0.12	0.31	0.15
EA vs. EC	2.39	0.03	0.52	0.03
EA vs. ED	3.35	0.03	0.97	0.03
EA vs. PA	2.77	0.03	0.96	0.03
EA vs. PB	3.59	0.03	0.95	0.03
EA vs. PC	1.44	0.04	0.48	0.03
EA vs. PD	2.48	0.03	0.73	0.02
EB vs. EC	2.34	0.03	0.42	0.03
EB vs. ED	3.34	0.03	0.79	0.03
EB vs. PA	2.97	0.03	0.78	0.04
EB vs. PB	3.68	0.04	0.89	0.04
EB vs. PC	1.46	0.03	0.47	0.04
EB vs. PD	2.07	0.06	0.54	0.06
EC vs. ED	2.37	0.03	0.60	0.03
EC vs. PA	1.76	0.02	0.49	0.03
EC vs. PB	1.98	0.03	0.44	0.03
EC vs. PC	1.17	0.08	0.24	0.06
EC vs. PD	1.45	0.10	0.22	0.11
ED vs. PA	2.54	0.03	0.92	0.03
ED vs. PB	2.49	0.02	0.84	0.02
ED vs. PC	1.14	0.03	0.22	0.03
ED vs. PD	2.93	0.04	0.74	0.03
PA vs. PB	1.89	0.03	0.54	0.03
PA vs. PC	1.16	0.07	0.26	0.07
PA vs. PD	2.11	0.07	0.56	0.03
PB vs. PC	1.12	0.07	0.25	0.02
PB vs. PD	2.16	0.03	0.58	0.03
PC vs. PD	1.15	0.11	0.29	0.10

Note: ANOSIM, analysis of similarities; PERMANOVA, permutational multivariate analysis of variance, used to evaluate differences in bacterial community composition. Ctrl, control group; EA, EB, EC, and ED represent PE treatments at 0.1%, 0.5%, 1%, and 5%, respectively; PA, PB, PC, and PD represent PS treatments at 0.1%, 0.5%, 1%, and 5%.

## Data Availability

The original contributions presented in this study are included in the article. Further inquiries can be directed to the corresponding author.

## References

[1] Geyer R., Jambeck J.R., Law K.L. (2017). Production, use, and fate of all plastics ever made. Sci. Adv..

[2] Thompson R.C., Olsen Y., Mitchell R.P., Davis A., Rowland S.J., John A.W., McGonigle D., Russell A.E. (2004). Lost at sea: Where is all the plastic?. Science.

[3] Bakir A., Rowland S.J., Thompson R.C. (2014). Enhanced desorption of persistent organic pollutants from microplastics under simulated physiological conditions. Environ. Pollut..

[4] Mammo F.K., Amoah I.D., Gani K.M., Pillay L., Ratha S.K., Bux F., Kumari S. (2020). Microplastics in the environment: Interactions with microbes and chemical contaminants. Sci. Total Environ..

[5] Klein M., Fischer E.K. (2019). Microplastic abundance in atmospheric deposition within the Metropolitan area of Hamburg, Germany. Sci. Total Environ..

[6] Ng E.L., Huerta Lwanga E., Eldridge S.M., Johnston P., Hu H.W., Geissen V., Chen D. (2018). An overview of microplastic and nanoplastic pollution in agroecosystems. Sci. Total Environ..

[7] Diao T., Liu R., Meng Q., Sun Y. (2022). Microplastics derived from polymer-coated fertilizer altered soil properties and bacterial community in a Cd-contaminated soil. Appl. Soil. Ecol..

[8] Huang D., Wang X., Yin L., Chen S., Tao J., Zhou W., Chen H., Zhang G., Xiao R. (2022). Research progress of microplastics in soil-plant system: Ecological effects and potential risks. Sci. Total Environ..

[9] Akbaş M., Babur E., Tüfekçioğlu A. (2026). Soil Physicochemical and Biochemical Differentiation Under Dominant Broadleaf Forest Species in the Eastern Black Sea Region. Forests.

[10] Zhang M., Zhao Y., Qin X., Jia W., Chai L., Huang M., Huang Y. (2019). Microplastics from mulching film is a distinct habitat for bacteria in farmland soil. Sci. Total Environ..

[11] Zettler E.R., Mincer T.J., Amaral-Zettler L.A. (2013). Life in the “plastisphere”: Microbial communities on plastic marine debris. Environ. Sci. Technol..

[12] Aralappanavar V.K., Mukhopadhyay R., Yu Y., Liu J., Bhatnagar A., Praveena S.M., Li Y., Paller M., Adyel T.M., Rinklebe J. (2024). Effects of microplastics on soil microorganisms and microbial functions in nutrients and carbon cycling—A review. Sci. Total Environ..

[13] Zhang X., Li Y., Ouyang D., Lei J., Tan Q., Xie L., Li Z., Liu T., Xiao Y., Farooq T.H. (2021). Systematical review of interactions between microplastics and microorganisms in the soil environment. J. Hazard. Mater..

[14] Dhevagi P., Keerthi Sahasa R.G., Poornima R., Ramya A. (2024). Unveiling the effect of microplastics on agricultural crops—A review. Int. J. Phytoremediat..

[15] Yi M., Zhou S., Zhang L., Ding S. (2021). The effects of three different microplastics on enzyme activities and microbial communities in soil. Water Environ. Res..

[16] Xu G., Liu Y., Yu Y. (2021). Effects of polystyrene microplastics on uptake and toxicity of phenanthrene in soybean. Sci. Total Environ..

[17] Meng F., Harkes P., van Steenbrugge J.J.M., Geissen V. (2023). Effects of microplastics on common bean rhizosphere bacterial communities. Appl. Soil. Ecol..

[18] Rillig M.C. (2018). Microplastic Disguising As Soil Carbon Storage. Environ. Sci. Technol..

[19] Zhou J., Gui H., Banfield C.C., Wen Y., Zang H., Dippold M.A., Charlton A., Jones D.L. (2021). The microplastisphere: Biodegradable microplastics addition alters soil microbial community structure and function. Soil. Biol. Biochem..

[20] Cai L., Zhao X., Liu Z., Han J. (2023). The abundance, characteristics and distribution of microplastics (MPs) in farmland soil—Based on research in China. Sci. Total Environ..

[21] Yan P., Zhang S., Wang J., Wang W., Xu B., Hao X., Aurangzeib M. (2022). Field management changes the distribution of mesoplastic and macroplastic in Mollisols of Northeast China. Chemosphere.

[22] Liu X., Zhou D.D., Chen M., Cao Y.W., Zhuang L.Y., Lu Z.H., Yang Z.H. (2022). Adsorption behavior of azole fungicides on polystyrene and polyethylene microplastics. Chemosphere.

[23] Sun Y., Duan C., Cao N., Li X., Li X., Chen Y., Huang Y., Wang J. (2022). Effects of microplastics on soil microbiome: The impacts of polymer type, shape, and concentration. Sci. Total Environ..

[24] Rong L., Zhao L., Zhao L., Cheng Z., Yao Y., Yuan C., Wang L., Sun H. (2021). LDPE microplastics affect soil microbial communities and nitrogen cycling. Sci. Total Environ..

[25] Hu X., Wang Y., Gu H., Liu J., Liu Z., Li Y., Jin J., Wang G. (2024). Changes in soil microbial functions involved in the carbon cycle response to conventional and biodegradable microplastics. Appl. Soil. Ecol..

[26] Chang S., Chen C., Fu Q.L., Zhou A., Hua Z., Zhu F., Li S., He H. (2024). PBAT biodegradable microplastics enhanced organic matter decomposition capacity and CO(2) emission in soils with and without straw residue. J. Hazard. Mater..

[27] Trevaskis B., Harris F.A.J., Bovill W.D., Rattey A.R., Khoo K.H.P., Boden S.A., Hyles J. (2022). Advancing understanding of oat phenology for crop adaptation. Front. Plant Sci..

[28] Gong M., Yang G., Zhuang L., Zeng E.Y. (2019). Microbial biofilm formation and community structure on low-density polyethylene microparticles in lake water microcosms. Environ. Pollut..

[29] Lee S.H., Sorensen J.W., Grady K.L., Tobin T.C., Shade A. (2017). Divergent extremes but convergent recovery of bacterial and archaeal soil communities to an ongoing subterranean coal mine fire. ISME J..

[30] Chen H., Wang Y., Sun X., Peng Y., Xiao L. (2020). Mixing effect of polylactic acid microplastic and straw residue on soil property and ecological function. Chemosphere.

[31] Zhuang M., Qiao C., Han L., Bi Y., Cao M., Wang S., Guo L., Pang R., Xie H. (2024). Multi-omics analyses reveal the responses of wheat (*Triticum aestivum* L.) and rhizosphere bacterial community to nano(micro)plastics stress. J. Nanobiotechnol..

[32] Maity S., Guchhait R., Sarkar M.B., Pramanick K. (2022). Occurrence and distribution of micro/nanoplastics in soils and their phytotoxic effects: A review. Plant Cell Environ..

[33] Saarma K., Tarkka M.T., Itävaara M., Fagerstedt K.V. (2003). Heat shock protein synthesis is induced by diethyl phthalate but not by di(2-ethylhexyl) phthalate in radish (*Raphanus sativus*). J. Plant Physiol..

[34] Zang H., Zhou J., Marshall M.R., Chadwick D.R., Wen Y., Jones D.L. (2020). Microplastics in the agroecosystem: Are they an emerging threat to the plant-soil system?. Soil. Biol. Biochem..

[35] Zhu F., Yan Y., Doyle E., Zhu C., Jin X., Chen Z., Wang C., He H., Zhou D., Gu C. (2022). Microplastics altered soil microbiome and nitrogen cycling: The role of phthalate plasticizer. J. Hazard. Mater..

[36] Qi Y., Ossowicki A., Yang X., Huerta Lwanga E., Dini-Andreote F., Geissen V., Garbeva P. (2020). Effects of plastic mulch film residues on wheat rhizosphere and soil properties. J. Hazard. Mater..

[37] Wang F., Zhang X., Zhang S., Zhang S., Sun Y. (2020). Interactions of microplastics and cadmium on plant growth and arbuscular mycorrhizal fungal communities in an agricultural soil. Chemosphere.

[38] Wang Y., Cheng H., Li Y., Ning R., Lv Y., Wang Q., Zhang H., Liu N. (2024). Individual and Combined Effects of Nanoplastics and Cadmium on the Rhizosphere Bacterial Community of Sedum alfredii Hance. Microorganisms.

[39] Fei Y., Huang S., Zhang H., Tong Y., Wen D., Xia X., Wang H., Luo Y., Barceló D. (2020). Response of soil enzyme activities and bacterial communities to the accumulation of microplastics in an acid cropped soil. Sci. Total Environ..

[40] Li K., He Q., Zhu J., Wang J., Sun C., Tan A., Zhao X., Peng Y., Huang C., Cai J. (2025). Responses of microbial communities to the addition of different types of microplastics in agricultural soils. Environ. Pollut..

[41] Sun Y., Ren X., Rene E.R., Wang Z., Zhou L., Zhang Z., Wang Q. (2021). The degradation performance of different microplastics and their effect on microbial community during composting process. Bioresour. Technol..

[42] Negi R., Sharma B., Kaur T., Renuka Jyothi S., Gupta A., Thakur N., Jhamta S., Rathore S., Yadav N., Kapoor M. (2026). Pseudomonadota: Biodiversity, functional annotation for plant growth and biotechnological applications for agro-environmental sustainability. Ecol. Front..

[43] Philippot L., Raaijmakers J.M., Lemanceau P., van der Putten W.H. (2013). Going back to the roots: The microbial ecology of the rhizosphere. Nat. Rev. Microbiol..

[44] Liu W., Wang Y., Gu C., Wang J., Dai Y., Maryam B., Chen X., Yi X., Liu X. (2025). Polyethylene microplastics distinctly affect soil microbial community and carbon and nitrogen cycling during plant litter decomposition. J. Environ. Manag..

[45] Feng X., Wang Q., Sun Y., Zhang S., Wang F. (2022). Microplastics change soil properties, heavy metal availability and bacterial community in a Pb-Zn-contaminated soil. J. Hazard. Mater..

[46] Zhai X., Zhang X.H., Yu M. (2023). Microbial colonization and degradation of marine microplastics in the plastisphere: A review. Front. Microbiol..

[47] Rillig M.C., Kim S.W., Zhu Y.-G. (2024). The soil plastisphere. Nat. Rev. Microbiol..

[48] Liang R., Sun F., Zhang C., Zhang R., Wang H., Wang X. (2023). Interaction between microplastics and microorganisms in soil environment: A review. Sheng Wu Gong Cheng Xue Bao.

[49] Takaichi S., Maoka T., Takasaki K., Hanada S. (2010). Carotenoids of Gemmatimonas aurantiaca (Gemmatimonadetes): Identification of a novel carotenoid, deoxyoscillol 2-rhamnoside, and proposed biosynthetic pathway of oscillol 2,2′-dirhamnoside. Microbiology.

[50] Qian H., Zhang M., Liu G., Lu T., Qu Q., Du B., Pan X. (2018). Effects of Soil Residual Plastic Film on Soil Microbial Community Structure and Fertility. Water Air Soil. Poll..

[51] Xiao M., Ding J., Luo Y., Zhang H., Yu Y., Yao H., Zhu Z., Chadwick D.R., Jones D., Chen J. (2022). Microplastics shape microbial communities affecting soil organic matter decomposition in paddy soil. J. Hazard. Mater..

[52] Zhang Z., Yang Z., Yue H., Xiao M., Ge T., Li Y., Yu Y., Yao H. (2022). Discrepant impact of polyethylene microplastics on methane emissions from different paddy soils. Appl. Soil. Ecol..

[53] Chen T., Shi Y., Peng C., Tang L., Chen Y., Wang T., Wang Z., Wang S., Li Z. (2022). Transcriptome Analysis on Key Metabolic Pathways in Rhodotorula mucilaginosa Under Pb(II) Stress. Appl. Environ. Microbiol..

[54] Wang K., Wang F., Yu Y., Yang S., Han Y., Yao H. (2025). Microplastics and soil microbiomes. BMC Biol..

[55] Chen Y., Li Y., Liang X., Lu S., Ren J., Zhang Y., Han Z., Gao B., Sun K. (2024). Effects of microplastics on soil carbon pool and terrestrial plant performance. Carbon Res..

[56] Yu H., Fan P., Hou J., Dang Q., Cui D., Xi B., Tan W. (2020). Inhibitory effect of microplastics on soil extracellular enzymatic activities by changing soil properties and direct adsorption: An investigation at the aggregate-fraction level. Environ. Pollut..

[57] Liu H., Yang X., Liu G., Liang C., Xue S., Chen H., Ritsema C.J., Geissen V. (2017). Response of soil dissolved organic matter to microplastic addition in Chinese loess soil. Chemosphere.

[58] Huang Y., Zhao Y., Wang J., Zhang M., Jia W., Qin X. (2019). LDPE microplastic films alter microbial community composition and enzymatic activities in soil. Environ. Pollut..

[59] Isaac P., Martínez F.L., Bourguignon N., Sánchez L.A., Ferrero M.A. (2015). Improved PAHs removal performance by a defined bacterial consortium of indigenous Pseudomonas and actinobacteria from Patagonia, Argentina. Int. Biodeterior. Biodegrad..

[60] Woźniak-Karczewska M., Lisiecki P., Białas W., Owsianiak M., Piotrowska-Cyplik A., Wolko Ł., Ławniczak Ł., Heipieper H.J., Gutierrez T., Chrzanowski Ł. (2019). Effect of bioaugmentation on long-term biodegradation of diesel/biodiesel blends in soil microcosms. Sci. Total Environ..

[61] Luo D., Li Y., Yao H., Chapman S.J. (2022). Effects of different carbon sources on methane production and the methanogenic communities in iron rich flooded paddy soil. Sci. Total Environ..

[62] Luo D., Meng X., Zheng N., Li Y., Yao H., Chapman S.J. (2021). The anaerobic oxidation of methane in paddy soil by ferric iron and nitrate, and the microbial communities involved. Sci. Total Environ..

[63] Malyan S.K., Bhatia A., Kumar A., Gupta D.K., Singh R., Kumar S.S., Tomer R., Kumar O., Jain N. (2016). Methane production, oxidation and mitigation: A mechanistic understanding and comprehensive evaluation of influencing factors. Sci. Total Environ..

[64] Han L., Zhang B., Li D., Chen L., Feng Y., Xue L., He J., Feng Y. (2022). Co-occurrence of microplastics and hydrochar stimulated the methane emission but suppressed nitrous oxide emission from a rice paddy soil. J. Clean. Prod..

[65] Han L., Chen L., Li D., Ji Y., Feng Y., Feng Y., Yang Z. (2022). Influence of polyethylene terephthalate microplastic and biochar co-existence on paddy soil bacterial community structure and greenhouse gas emission. Environ. Pollut..

[66] Riveros G., Urrutia H., Araya J., Zagal E., Schoebitz M. (2022). Microplastic pollution on the soil and its consequences on the nitrogen cycle: A review. Environ. Sci. Pollut. Res. Int..

[67] Mahmud K., Makaju S., Ibrahim R., Missaoui A. (2020). Current Progress in Nitrogen Fixing Plants and Microbiome Research. Plants.

[68] Han L., Chen L., Feng Y., Kuzyakov Y., Chen Q., Zhang S., Chao L., Cai Y., Ma C., Sun K. (2024). Microplastics alter soil structure and microbial community composition. Environ. Int..

[69] Sun X., Tao R., Xu D., Qu M., Zheng M., Zhang M., Mei Y. (2023). Role of polyamide microplastic in altering microbial consortium and carbon and nitrogen cycles in a simulated agricultural soil microcosm. Chemosphere.

[70] Wang C., Zhao J., Xing B. (2021). Environmental source, fate, and toxicity of microplastics. J. Hazard. Mater..

[71] Hasan Anik A., Hossain S., Alam M., Binte Sultan M., Hasnine M.D.T., Rahman M.M. (2021). Microplastics pollution: A comprehensive review on the sources, fates, effects, and potential remediation. Environ. Nanotechnol. Monit. Manag..

[72] Meng F., Yang H., Fan X., Gao X., Tai J., Sa R., Ge X., Yang X., Liu Q. (2023). A microbial ecosystem enhanced by regulating soil carbon and nitrogen balance using biochar and nitrogen fertiliser five years after application. Sci. Rep..

[73] Sun Y., Wang C., Ruan H. (2022). Increased microbial carbon and nitrogen use efficiencies under drought stress in a poplar plantation. For. Ecol. Manag..

[74] Xiao S., Zhang H., Zhu R., Liao X., Wu Y., Mi J., Wang Y. (2021). Ammonia reduction by the gdhA and glnA genes from bacteria in laying hens. Ecotoxicol. Environ. Saf..

[75] de Souza Machado A.A., Lau C.W., Kloas W., Bergmann J., Bachelier J.B., Faltin E., Becker R., Görlich A.S., Rillig M.C. (2019). Microplastics Can Change Soil Properties and Affect Plant Performance. Environ. Sci. Technol..

[76] Palansooriya K.N., Sang M.K., El-Naggar A., Shi L., Chang S.X., Sung J., Zhang W., Ok Y.S. (2023). Low-density polyethylene microplastics alter chemical properties and microbial communities in agricultural soil. Sci. Rep..

[77] Zhang H., Huang Y., An S., Zhu Z. (2022). A Review of Microplastics in Soil: Distribution Within Pedosphere Compartments, Environmental Fate, and Effects. Water Air Soil Pollut..

[78] Gaby J.C., Buckley D.H. (2014). A comprehensive aligned nifH gene database: A multipurpose tool for studies of nitrogen-fixing bacteria. Database.

[79] Zehr J.P., Turner P.J., Whitman W.B. (2001). Nitrogen fixation: Nitrogenase genes and gene expression. Methods in Microbiology.

[80] Su J., Jin L., Jiang Q., Sun W., Zhang F., Li Z. (2013). Phylogenetically diverse ureC genes and their expression suggest the urea utilization by bacterial symbionts in marine sponge Xestospongia testudinaria. PLoS ONE.

[81] Wang F., Wang Q., Adams C.A., Sun Y., Zhang S. (2022). Effects of microplastics on soil properties: Current knowledge and future perspectives. J. Hazard. Mater..

[82] Sakoula D., Smith G.J., Frank J., Mesman R.J., Kop L.F.M., Blom P., Jetten M.S.M., van Kessel M., Lücker S. (2022). Universal activity-based labeling method for ammonia- and alkane-oxidizing bacteria. ISME J..

[83] Wright C.L., Schatteman A., Crombie A.T., Murrell J.C., Lehtovirta-Morley L.E. (2020). Inhibition of Ammonia Monooxygenase from Ammonia-Oxidizing Archaea by Linear and Aromatic Alkynes. Appl. Environ. Microbiol..

[84] Wang M., Li D., Liu X., Chen C., Frey B., Sui X., Li M.H. (2024). Microplastics stimulated soil bacterial alpha diversity and nitrogen cycle: A global hierarchical meta-analysis. J. Hazard. Mater..

[85] Yang Z., Zhao L., Tan S., Mao P., Wang Q., Ma W. (2025). Effects of Polyethylene and Polystyrene Microplastics on Oat (*Avena sativa* L.) Growth and Physiological Characteristics. Plants.

[86] Zhou J., Bruns M.A., Tiedje J.M. (1996). DNA recovery from soils of diverse composition. Appl. Environ. Microbiol..

[87] Caporaso J.G., Lauber C.L., Walters W.A., Berg-Lyons D., Huntley J., Fierer N., Owens S.M., Betley J., Fraser L., Bauer M. (2012). Ultra-high-throughput microbial community analysis on the Illumina HiSeq and MiSeq platforms. ISME J..

[88] Callahan B.J., McMurdie P.J., Rosen M.J., Han A.W., Johnson A.J., Holmes S.P. (2016). DADA2: High-resolution sample inference from Illumina amplicon data. Nat. Methods.

[89] Bokulich N.A., Kaehler B.D., Rideout J.R., Dillon M., Bolyen E., Knight R., Huttley G.A., Gregory Caporaso J. (2018). Optimizing taxonomic classification of marker-gene amplicon sequences with QIIME 2’s q2-feature-classifier plugin. Microbiome.

[90] Mandal S., Van Treuren W., White R.A., Eggesbo M., Knight R., Peddada S.D. (2015). Analysis of composition of microbiomes: A novel method for studying microbial composition. Microb. Ecol. Health Dis..

[91] Oksanen J., Blanchet F.G., Friendly M., Kindt R., Legendre P., McGlinn D., Minchin P.R., O’Hara R.B., Simpson G.L., Solymos P. (2020). vegan: Community Ecology Package.

[92] Vázquez-Baeza Y., Pirrung M., Gonzalez A., Knight R. (2013). EMPeror: A tool for visualizing high-throughput microbial community data. Gigascience.

[93] Segata N., Izard J., Waldron L., Gevers D., Miropolsky L., Garrett W.S., Huttenhower C. (2011). Metagenomic biomarker discovery and explanation. Genome Biol..

[94] Langille M.G., Zaneveld J., Caporaso J.G., McDonald D., Knights D., Reyes J.A., Clemente J.C., Burkepile D.E., Vega Thurber R.L., Knight R. (2013). Predictive functional profiling of microbial communities using 16S rRNA marker gene sequences. Nat. Biotechnol..

